# Patient Satisfaction with Virtual Clinic Encounters: Analysis of Factors that Impact the Press Ganey Survey in the Hand Surgery Population

**DOI:** 10.1016/j.jhsg.2023.02.004

**Published:** 2023-03-07

**Authors:** Miranda J. Rogers, Matthew Belton, Dustin Randall, Minkyoung Yoo, Angela P. Presson, Angela Wang, Nikolas H. Kazmers

**Affiliations:** ∗ Department of Orthopaedic Surgery, University of Utah, Salt Lake City, UT; † Department of Economics, University of Utah, Salt Lake City, UT; ‡ Division of Public Health, University of Utah, Salt Lake City, UT

**Keywords:** Patient satisfaction, Press Ganey, Virtual visits, Patient factors

## Abstract

**Purpose:**

Our purpose was to identify patient characteristics and visit components that affect patient satisfaction with virtual new patient visits in an outpatient hand surgery clinic as measured by the Press Ganey Outpatient Medical Practice Survey (PGOMPS) total score (primary outcome) and provider subscore (secondary outcome).

**Methods:**

Adult patients evaluated through virtual new patient visits at a tertiary academic medical center between January 2020 and October 2020 who completed the PGOMPS for virtual visits were included. Data regarding demographics and visit characteristics were collected via chart review. Factors associated with satisfaction were identified using a Tobit regression model on the continuous score outcomes (Total Score and Provider Subscore) to account for substantial ceiling effects.

**Results:**

A total of 95 patients were included: 54% were men and the mean age was 54 ± 16 years. Mean area deprivation index was 32 ± 18, and the mean driving distance to the clinic was 97 ± 188 mi. Common diagnoses include compressive neuropathy (21%), hand arthritis (19%), hand mass (12%), and fracture/dislocation (11%). Treatment recommendations included small joint injection (20%), in-person evaluation (25%), surgery (36%), and splinting (20%). Multivariable Tobit regressions showed notable differences in satisfaction by the provider on the Total Score but not on the Provider Subscore. Other factors known to affect the PGOMPS scores for in-person visits were not notably associated with the Total or Provider Sub-Scores for virtual visits (area deprivation index, age, and offer of surgery or injection) other than the body mass index.

**Conclusions:**

Virtual clinic visit satisfaction was affected by the provider. Wait time strongly affects satisfaction with in-person visits but is not accounted for by the PGOMPS scoring system for virtual visits, which is a limitation of their survey. Further work is required to determine how to improve the patient experience with virtual visits.

**Type of study/level of evidence:**

Prognostic IV.

In the past decade, institutions have begun measuring patient satisfaction with physician encounters.^1^^`^ The Press Ganey Outpatient Medical Practice Survey (PGOMPS) is a commonly used performance metric with the ability to assess multiple aspects of the patient outpatient experience,^2^ from wait times and facility resources to the quality of interaction with the physician and staff.^3^ Previous studies have indicated that increased age,^4^ decreased wait times,^5^ and receiving an intervention^6^ have been associated with increased patient satisfaction for new outpatient visits. The topic is complicated, and satisfaction with the clinical process and satisfaction with the final outcome may differ.^7^ Assessment of patient satisfaction has become an integral part of measuring and tracking patient care,^2^^,^^7^^,^^8^ with an ongoing discussion about the merits and challenges of these metrics and concerns that it may not correlate closely with surgical satisfaction or outcomes and unnecessarily impact medical institutions.^9^

Patient interfaces within the health care system have changed over time. With the increased prevalence of broadband internet connection across the country, some providers have offered patients telemedicine encounters in lieu of an in-person visit. This has historically been particularly useful in connecting patients in socially deprived or medically isolated regions; nevertheless, telemedicine use surged in response to restrictions placed by the recent COVID-19^10^ pandemic in both the rural and urban settings.^11^ Within the hand and upper-extremity surgery, the transition to virtual encounters can be wrought with obstacles that may affect patient satisfaction; nevertheless, patients are generally accepting of the approach.^12^, ^13^, ^14^ Given the demand for telemedicine, the Press Ganey Corporation has adjusted their metrics by streamlining the questions to 12 total across four domains, with the pertinent addition of “Telemedicine Technology” section.^15^ Telemedicine continues to be in use, even with the return of in-person visits, which speaks to the importance of understanding how it influences patient care. Defining patient and visit characteristics predictive of high encounter satisfaction in a virtual setting provides opportunities to enhance future clinical experiences and increase the effectiveness of patient outreach. The purpose of this study was to identify the patient characteristics and/or visit components that affect patient satisfaction with virtual new patient visits (NPVs) in an outpatient hand surgery clinic as measured by the PGOMPS Total Score (primary outcome) and Provider Subscore (secondary outcome).

## Materials and Methods

In this institutional review board-approved study, all adult patients (**≥**18 years old) who were evaluated by six hand surgery providers (4 surgeons and one physician assistant) between January 2020 and October 2020 for virtual NPV driven by COVID-19 pandemic–related precautions at a single tertiary academic medical center were considered for inclusion pending completion of the survey. Exclusion criteria included patients <18 years old, those without corresponding video visits, and those with a virtual follow-up visit instead of NPV.

At our institution, all clinic encounters for hand and upper-extremity patients included an automatic email survey link proctored by the Press Ganey Corporation following their clinic appointment. A second email is sent in 5 days if patients do not respond to the initial survey. The survey link is available for 30 days following the clinic visit. Generally, the PGOMPS consists of 24 questions grouped into six domains that evaluate an individual patient’s perception of several aspects of health care delivery in the outpatient setting.^16^ In addition, PGOMPS contains a Telemedicine Technology section.^15^ Each question offers a Likert scale ranging from 1 (very poor) to 5 (very good). The scores are calculated using the equations proprietary to the Press Ganey Corporation and reported to the using institution. They are stratified by the Total Score and Provider Subscore, with a higher score denoting a higher level of patient satisfaction.

A retrospective chart review collected the demographic variables, including sex, race/ethnicity, body mass index (BMI), area deprivation index (ADI), insurance type, smoking status, and primary diagnosis. Visit characteristics were also collected, such as distance from the clinic and the treatments offered (surgery, injections, advanced imaging, or in-person clinic visit recommendation). ADI is a measure calculated by the Health Resources and Services Administration and adapted to the Census by researchers at the University of Wisconsin-Madison.^17^^,^^18^ The index ranks a neighborhood, defined by their Zip+4 code, encompassing approximately 10 to 20 homes, by their theoretical disadvantage as measured by 17 different factors such as average income, education level, employment percentage, and housing. ADI rankings comprise both state-only deciles and national percentiles, and higher rankings represent areas with increased social deprivation. We also evaluated which interventions were recommended and whether an in-person visit was recommended to allow for additional clinical evaluation.

Summary statistics of patient demographics, clinical characteristics, and outcomes were provided for the overall study cohort. Mean with standard deviation and median with interquartile range were provided for continuous outcome variables, and frequency with percentage was provided for categorical variables. Univariate and multivariable regression analyses were performed to identify the factors determining the PGOMPS Total Score using the Tobit model accounting the high ceiling effect.^19^^,^^20^ Potential predictors included in the model were age, sex, race/ethnicity, insurance status, smoking status, provider, diagnosis (arthritis, Dupuytren disease, fracture-dislocation, mass, traumatic nerve, pain, soft tissue traumatic, tendinitis, and others), and treatment (injection, surgery, in-person evaluation, advanced imaging, splinting, therapy, and follow-up as needed). Smoking status and BMI were recategorized because of a small sample size in certain categories. Similar analyses were performed using the PGOMPS Provider Subscore. All statistical tests were evaluated at a two-sided α = 0.05 level.

## Results

After excluding patients who were <18 years old (2), those without corresponding video visits (11), and those with virtual follow-up visits rather than an NPV (27), a total of 95 new hand patients were included (70% inclusion). Mean age was 54 ± 16 years; 54% (52) were men, and 87% (83) were identified as White (non-Hispanic). Most (62%) had commercial insurance, and 65% never smoked. Approximately two-thirds of the patients were overweight or obese (34% overweight and 41% obese). Primary diagnoses included compressive neuropathy (21%), hand arthritis (19%), fracture/dislocation (11%), hand mass (12%), and Dupuytren disease (6%). Common treatment recommendations included a small joint injection (20%), in-person evaluation to further elucidate the pathology (25%), surgery (36%), and splinting (20%). The average national percentile for ADI was 32 ± 18 with the average distance to a nearby clinic being 97 ± 188 miles (Table 1).

**Table 1:** Baseline Patient Characteristics and Demographics

Patient Demographic Factors†	Mean ± SD or Count (%)	Median (IQR)
Age (y)	54 (16)	55 (23)
Driving distance (miles)	97 (188)	22 (48)
ADI (national percentile)	32 (18)	29 (27)
Insurance		
Commercial	59 (62)	
Medicaid/Other Government	12 (13)	
Medicare	24 (25)	
Gender (man)	52 (55)	
Race/Ethnicity		
White/Caucasian (Non-Hispanic)	83 (87)	
Other/Unknown	12 (13)	
Smoking status∗		
Never smoker	65 (68)	
Former smoker	22 (23)	
Current smoker	5 (5)	
Provider		
A	26 (27)	
B	11 (12)	
C	9 (9)	
D	39 (41)	
E	10 (11)	
BMI		
Underweight	1 (1)	
Normal	17 (18)	
Overweight	32 (34)	
Obese	39 (41)	
Unknown	6 (6)	
**Diagnostic and Treatment Factors**		
Diagnosis		
Arthritis	18 (19)	
Dupuytren disease	6 (6)	
Fracture-dislocation	10 (11)	
Mass	11 (12)	
Compressive neuropathy	20 (21)	
Pain not otherwise specified	14 (15)	
Soft tissue traumatic	3 (3)	
Tendinitis	5 (5)	
Other†	8 (8)	
Treatment		
Injection recommended/performed	19 (20)	
Surgeryrecommended/performed	34 (36)	
In-person evaluation	24 (25)	
Advanced imaging	10 (11)	
Splinting	19 (20)	
Therapy	5 (5)	
Follow -up as needed	21 (22)	
**Outcomes: PGOMPS Scores**		
PGOMPS Total Score	84 (20)	93 (29)
PGOMPS Provider Subscore	88 (22)	100 (20)

∗ N = 92 (3 patients did not report smoking status).

† N = 95.

For the included patients, the PGOMPS Total Score was 84 ± 20 and the Subscore was 88 ± 22. Univariate and multivariable regression analyses were performed to identify the patient factors influencing the PGOMPS Total Score (Table 2). According to the results from univariate analyses, race/ethnicity and recommendation of an in-person evaluation were potentially associated with a decreased Total Score, whereas the provider influenced the overall Total Score. After performing a multivariable analysis, we found that certain providers were notably associated with a higher PGOMPS Total Score. In addition, meeting an overweight BMI classification was also notably associated with a higher PGOMPS Total Score.

**Table 2:** Predictors of Satisfaction on the Press Ganey Total Score

Patient Demographic Factors	Univariable Analysis†		Multivariable Analysis†	
	Coefficient (95% CI)	*P* value	Coefficient (95% CI)	*P* value
Age (y)	0.21 (−0.03, 0.45)	0.092	−0.05 (−0.64, 0.53)	0.857
Driving distance (miles)	0 (−0.02, 0.02)	0.815	0.02 (−0.02, 0.06)	0.341
ADI (national percentile)	−0.14 (−0.36, 0.08)	0.221	−0.28 (−0.7, 0.15)	0.201
Insurance				
Commercial	Reference		Reference	
Medicaid/Other Government	−5.74 (−18.01, 6.53)	0.355	−1.54 (−23.9, 20.81)	0.891
Medicare	1.98 (−7.4, 11.36)	0.676	6.39 (−12.06, 24.84)	0.491
Gender (man)	0.43 (−7.6, 8.47)	0.915	7.84 (−4.98, 20.67)	0.226
Race/Ethnicity (Caucasian)				
White/Caucasian (Non-Hispanic)	Reference		Reference	
Other/Unknown	−12.17 (−23.96, −0.39)	0.043	−11.73 (−33.1, 9.65)	0.277
Smoking status∗				
Never smoker	Reference		Reference	
Former or Current smoker	4.48 (−4.4, 13.36)	0.319	14.64 (−2.81, 32.08)	0.098
BMI				
Underweight or Normal	Reference		Reference	
Overweight	6.04 (−5.38, 17.47)	0.296	21.24 (1.66, 40.81)	0.034
Obese	4.19 (−6.86, 15.24)	0.453	16.1 (−1.67, 33.86)	0.075
Unknown	2.63 (−15.65, 20.91)	0.776	9.17 (−22.92, 41.27)	0.570
**Diagnostic and Treatment Factors**				
Provider				
A	Reference		Reference	
B	15.68 (2.16, 29.2)	0.023	23.21 (1.02, 45.4)	0.041
C	11.46 (−3.08, 25.99)	0.121	12.72 (−11.84, 37.29)	0.305
D	10.34 (0.83, 19.86)	0.034	9.59 (−5.58, 24.76)	0.211
E	9.06 (−4.92, 23.05)	0.201	23.92 (−1.14, 48.97)	0.061
Diagnostic Category				
Arthritis	Reference		Reference	
Dupuytren disease	2.81 (−15.2, 20.82)	0.757	19.1 (−12.72, 50.92)	0.235
Fracture-dislocation	2.16 (−12.91, 17.23)	0.776	3.95 (−23.27, 31.17)	0.773
Mass	−0.53 (−15.15, 14.09)	0.942	−3.06 (−30.76, 24.65)	0.826
Compressive neuropathy	0.89 (−11.52, 13.3)	0.887	−3.3 (−25.14, 18.54)	0.764
Pain not otherwise specified	−1.39 (−15.01, 12.22)	0.839	−2.71 (−26.44, 21.02)	0.820
Soft tissue traumatic	−16.35 (−40.18, 7.47)	0.176	−22.49 (−55.35, 10.38)	0.176
Tendinitis	−7.8 (−27.11, 11.51)	0.424	−4.57 (−33.18, 24.03)	0.750
Other	−7.57 (−23.8, 8.66)	0.357	−9.53 (−35.95, 16.89)	0.473
Treatment				
Injection recommended/performed	−2.49 (−12.48, 7.5)	0.622	4.84 (−16.53, 26.21)	0.652
Surgery recommended/performed	7.44 (−0.77, 15.65)	0.075	8.29 (−15.2, 31.77)	0.483
In−person evaluation	−10.33 (−19.29, −1.36)	0.024	−13.1 (−34.34, 8.15)	0.223
Advanced imaging	−5.72 (−18.71, 7.26)	0.384	5.78 (−15.7, 27.25)	0.593
Splinting	0.86 (−9.43, 11.14)	0.869	10.81 (−6.29, 27.91)	0.211
Therapy	3.71 (−14.71, 22.13)	0.690	6.39 (−23.72, 36.5)	0.673
Follow-up PRN	0.18 (−9.46, 9.83)	0.970	3.18 (−18.77, 25.13)	0.773

∗ N = 92.

† N = 95.

Univariate and multivariable regression analyses were also performed to identify the patient factors influencing the PGOMPS Provider Subscore (Table 3). Univariate analyses found that the provider was potentially associated with a higher Subscore. After controlling this in a multivariable analysis, no factors were notably associated with the PGOMPS Provider Subscore.

**Table 3:** Predictors of Satisfaction on the Press Ganey Provider Subscore

Patient Demographic Factors	Univariable Analysis†		Multivariable Analysis†	
	Coefficient (95% CI)	*P* value	Coefficient (95% CI)	*P* value
Age (y)	−0.11 (−0.69, 0.48)	.718	0.56 (−0.45, 1.57)	.272
Driving distance (miles)	0.02 (−0.02, 0.06)	.339	0.01 (−0.07, 0.08)	.882
ADI (national percentile)	−0.3 (−0.71, 0.12)	.160	−0.16 (−0.9, 0.58)	.669
Insurance				
Commercial	Reference		Reference	
Medicaid/Other Government	−9.43 (−22.91, 4.06)	.168	1.73 (−36.91,40.36)	.929
Medicare	2.86 (−7.45, 13.17)	.583	4.89 (−26.06,35.84)	.753
Gender (man)	7.08 (−5.32, 19.48)	.258	8.98 (−14.01,31.96)	.438
Race/Ethnicity (Caucasian)				
White/Caucasian (Non-Hispanic)	Reference		Reference	
Other/Unknown	−14.56 (−36.54, 7.43)	.190	−15.59 (−50.81, 19.62)	.379
Smoking status∗				
Never smoker	Reference		Reference	
Former or current smoker	0.46 (−9.42, 10.35)	.926	7.84 (−21.67, 37.35)	.597
BMI				
Underweight or Normal/healthy weight	Reference		Reference	
Overweight	−0.19 (−12.88, 12.5)	.976	18.08 (−15.89, 52.05)	.291
Obese	−2.32 (−14.59, 9.95)	.708	12.29 (−18.22, 42.8)	.424
Unknown	4.03 (−16.28, 24.33)	.695	32.41 (−27.1, 91.91)	.281
**Diagnostic and Treatment Factors**				
Provider				
A	Reference		Reference	
B	22.58 (0.7, 44.46)	.043	8.9 (−28.12, 45.93)	.632
C	16.23 (−7.79, 40.26)	.182	28.31 (−20.25, 76.86)	.248
D	11.63 (−3.62, 26.88)	.132	−9.37 (−36, 17.26)	.484
E	22.33 (−3.12, 47.78)	.084	20.25 (−22.39, 62.88)	.346
Diagnostic Category				
Arthritis	Reference		Reference	
Dupuytren disease	18.58 (−13.13, 50.29)	.246	8.12 (−45.29, 61.53)	.762
Fracture−dislocation	5.4 (−21.66, 32.46)	.691	30.91 (−20.17, 81.98)	.231
Mass	−4.69 (−31.6, 22.22)	.729	−16.22 (−62.03, 29.58)	.482
Compressive neuropathy	−5.35 (−27.31, 16.62)	.628	−1.94 (−39.62, 35.75)	.918
Pain not otherwise specified	−5.64 (−29.42, 18.14)	.637	14.88 (−25.9, 55.66)	.469
Soft tissue traumatic	−23.89 (−56.37, 8.59)	.147	−35.28 (−89.53, 18.98)	.199
Tendinitis	−9.09 (−37.59, 19.41)	.526	−5.33 (−54.04, 43.38)	.828
Other	−11.44 (−40.06, 17.18)	.427	−7.87 (−53.27, 37.52)	.730
Treatment				
Injection recommended/performed	7.44 (−13.78, 28.67)	.486	−12.19 (−47.86, 23.47)	.497
Surgery recommended/performed	8.47 (−15.01, 31.95)	.474	4.4 (−35, 43.79)	.824
In−person evaluation	−19.2 (−40.88, 2.48)	.082	−30.37 (−67.51, 6.77)	.107
Advanced imaging	12.37 (−9.69, 34.44)	.267	9.54 (−28.53, 47.62)	.618
Splinting	10.52 (−6.95, 27.99)	.233	1.46 (−28.06, 30.99)	.921
Therapy	12.89 (−17.28, 43.05)	.396	−10.65 (−60.6, 39.31)	.672
Follow-up PRN	3.74 (−18.12, 25.6)	.734	3.88 (−32.95, 40.71)	.834

∗ N = 92.

† N = 95.

## Discussion

Given the increasing use of telemedicine in health care, this study sought to identify the patient characteristics and/or visit components that affect patient satisfaction with virtual NPVs in a hand and upper-extremity clinic as documented by the PGOMS Total Score and Subscore. Both overweight BMI status and the surgeon providing care were associated with a notably higher Press Ganey Total Score. No patient covariates were notably associated with the Provider Subscore. Variables such as ADI, distance from the nearest clinic, age, and diagnosis did not statistically influence the patient satisfaction with virtual NPV.

During the COVID-19 pandemic, health care underwent a series of changes that led to increased usage of telemedicine—including virtual visits and telephone calls. Under the Coronavirus Preparedness and Response Supplemental Appropriations Act and Section 1135 waiver authority,^21^ the Centers for Medicare & Medicaid Services (CMS) expanded compensated virtual services to coincide with standard social distancing guidelines. Before this legislation, providers would only be compensated for a telehealth encounter if the patient met a strict set of criteria, initially designed to assist those in medically underserved areas, yet still requiring the patient to travel to designated medical facilities to receive the telehealth appointment. Now, all patients, regardless of diagnosis or location, could elect to receive their clinical care through telephone or virtual video appointments, limiting in-person encounters to urgent diagnoses or surgery.^22^^,^^23^ When properly applied, virtual visits have been found to be both cost efficient^24^ and diagnostically accurate.^25^ During the pandemic, virtual visits and the routine collection of Press Ganey metrics became the standard of care at our institution with satisfaction set *a priori* above the 33rd percentile threshold.^4^^,^^6^^,^^26^^,^^27^ Although there has been a gradual return to in-person visits, telemedicine remains in use in our practice and benefits patients who live at great distances from our institution. Even as the pandemic transitions, telemedicine is likely to remain an integral modality of health care delivery.^28^

Understanding the different variables and how it contributes to patient satisfaction within the clinical encounter has recently been of interest for provider and payers alike,^29^ with the PGOMPS being a reliable metric to capture these sentiments.^2^^,^^30^^,^^31^ Across the studies, these variables have been stratified by modifiable and non-modifiable patient-specific factors whose interplay shape the overall patient experience. As telemedicine becomes increasingly popular, both by demand and through increasing access to technology, many factors that we have largely understood to be impactful during in-person clinical encounters are no longer relevant. For example, modifiable factors such as longer wait times and time spent with provider were strongly associated with low patient satisfaction scores in the primary care setting.^32^ Recent publications show wait time as a strong negative predictor of satisfaction for in-person visits.^5^^,^^33^, ^34^, ^35^ Other studies suggest that wait time is often inaccurately perceived and often underestimated by patients—with the amount of time spent waiting being offset by actual time with the provider^36^ and provider-specific factors accounting for 80% of satisfactions scores.^16^

In addition, patient satisfaction has been found to be notably impacted by surgeon empathy, rather than visit duration or patient expectation of the visit length.^37^ Our findings did coincide with similar studies suggesting that the provider themselves^37^ and provider treatment recommendation(s) are a strong predictor of patient satisfaction.^6^^,^^38^^,^^39^ Specifically, the recommendation of an in-person visit was perceived negatively by patients compared with recommendations of surgery or advanced imaging. Patients tend to have a higher satisfaction with virtual visits during which they can clearly see and understand their provider.^40^ Taking all of these factors into account, a reasonable conclusion seems to be that patients still prioritize the same factors in virtual visits as in the clinic: empathetic discussions with their provider that are easy to understand. In a virtual setting, it becomes important for providers to use platforms that allow for clear communications with limited interruptions.

Factors such as increasing patient age, the offer of surgery or injection, and decreased wait time have a notable influence patient satisfaction in the hand surgery clinic setting.^6^ Non-modifiable risk factors such as sex, age, and economic status have also been associated with in-person visit patient satisfaction. In hand surgery spine clinics, research has shown that younger age, less formal education, and male sex are associated with lower patient satisfaction scores, whereas marital status, pain characteristics, and narcotic use were not influential.^41^ Increased satisfaction has been correlated with age in additional studies,^4^^,^^6^^,^^33^^,^^35^^,^^36^^,^^42^, ^43^, ^44^; nevertheless, it was not shown to influence satisfaction in our study. This speaks to the fact that no specific age group was notably impacted by virtual care delivery. Older patients have been varying satisfaction with virtual visits,^45^ although thoughtfully planned virtual visits that ensure patients are receiving acceptable care seems to ameliorate these concerns.^46^ In addition, recent research has shown that older patients were able to rapidly adopt and use digital technology during the pandemic.^47^ Our results did show that smoking status impacted satisfaction with virtual visits, conflicting with an earlier study of spine patients that revealed it to be correlated to diminished satisfaction.^41^ Comparatively, sex,^42^ social deprivation,^41^^,^^44^^,^^48^, ^49^, ^50^, ^51^, ^52^, ^53^, ^54^, ^55^ distance from clinic,^4^ and diagnosis were not found to affect patient satisfaction in the virtual setting, although they have been shown to be relevant in the in-person setting. Although overweight status was correlated with increased satisfaction, it is unclear why this specific BMI classification is of relevance. In addition, identifying why some variables no longer impact satisfaction is an area of interest that deserves additional research and cannot be commented on without speculation.

Socioeconomic data through the Neighborhood Atlas have provided additional factors that allow us to understand different predictors of satisfaction.^17^ Our findings did not suggest social deprivation or socioeconomic factors as being influential in virtual visit satisfaction scores, despite previous in-person studies showing an inverse relationship between less formal education,^41^^,^^51^ non-White race,^44^^,^^55^^,^^56^ insurance type,^48^ mental health,^48^ and socioeconomic status, as measured by the Area Deprivation Index^49^^,^^50^ with overall patient satisfaction and/or patient-reported outcomes (PROs). This differs from findings in orthopedics—and for medicine, in general—where higher deprivation is associated with lower satisfaction.^57^^,^^58^ It is possible that we are not powered to detect an association, or it is possible that by default, patients with smartphones and/or computers may be in a different category of social deprivation than the average patient. Insurance type was not found to be a predictor of satisfaction in our study, which is consistent with Rane et al^6^ but in contrast to what was documented by Tisano et al.^48^ It is possible that virtual visits level the playing field for patients who are socioeconomically disadvantaged and that patients with higher ADI are now able to be just as satisfied with virtual visits as those with lower ADI.

Finally, it is important to consider the fact that patient satisfaction can be multifaceted. Graham et al brought up the concept of dividing it into patient satisfaction with the clinical process (ie, the steps involved in attending and completing a clinical visit) versus satisfaction with the clinical outcome.^7^ Although these concepts may be interrelated, our current study focuses on understanding patient satisfaction with the clinical process. Further research is needed to assess how satisfaction with the experience of receiving clinical care relates to and influences ultimate satisfaction with the outcome. In addition, although the Press Ganey system is a commonly used performance metric that assesses multiple aspects of the patient outpatient experience, its relationship to satisfaction is debated and not fully understood. Kohring et al assessed the relationship between PGOMPS in 540 visits in patients undergoing primary total joint arthroplasty over a 3-year period, finding no correlation between patient satisfaction as determined by the Press Ganey score and patient perception of global health measures and physical function at 90 days and 1 year after surgery.^59^ Similarly, Chughtai et al documented no correlation between the Press Ganey Survey and commonly used total hip arthroplasty assessment tools (Harris Hip Score, Short Form-12 and Short Form-36, Hip Western Ontario and McMaster Universities Osteoarthritis Index, and the University of California Los Angeles and Visual Analog Scale scores) in a group of 692 patients who underwent total hip arthroplasty.^60^ This is important, given the potential for Press Ganey to be used by the Center of Medicare and Medicaid services,^60^ and for the fact that it highlights that satisfaction and outcome measures may run in parallel without being predictive of one another. In addition, surgeon performance, as assessed by Press Ganey, is based in part on the patient reporting of their experience. The fact that patient satisfaction and their outcome(s) are not always correlated should serve as a note of caution to those overly emphasizing either factor until that relationship is better understood. This is further magnified when incentive bonus payments based on provider performance for Medicare Advantage plans nearly quadrupled from $3 billion to $11.6 billion between 2015 and 2021.^61^

Limitations that warrant mention include the fact that we did not assess for patient mental health diagnosis because patients with documented depression have been shown to be less satisfied in the sports medicine clinic setting.^48^ In addition, we did not assess the pre-encounter levels of patient-reported physical function.^26^^,^^52^ Wait times were not recorded within the encounter because of the limitation of the virtual check-in interface. In the virtual setting, wait times could be considered more provider dependent, given the lack of external variables such as rooming and radiographs in the in-person setting, which could explain the variation in satisfaction between Total and Subscore. Confounders must also be considered, including the historically low response rate for the Press Ganey Survey, specifically with male sex, insurance type, and subspecialty encounters generating lower response rates.^3^^,^^62^^,^^63^ This is likely a flaw of the Press Ganey virtual visit survey design. Finally, an assessment of the quality of the virtual visit and its relationship to patient satisfaction would be an interesting next step as we continue to provide virtual clinical care. Notable strengths are the inclusion of ADI, given that socioeconomic deprivation has been notably correlated with hand trauma/injuries.^54^^,^^64^, ^65^, ^66^, ^67^
